# Effects of Lethal Bronzing Disease, Palm Height, and Temperature on Abundance and Monitoring of *Haplaxius crudus*

**DOI:** 10.3390/insects11110748

**Published:** 2020-10-30

**Authors:** De-Fen Mou, Chih-Chung Lee, Philip G. Hahn, Noemi Soto, Alessandra R. Humphries, Ericka E. Helmick, Brian W. Bahder

**Affiliations:** 1 Fort Lauderdale Research and Education Center, Department of Entomology and Nematology, University of Florida, 3205 College Ave., Ft. Lauderdale, FL 33314, USA; sn21377@ufl.edu (N.S.); ahumphries@ufl.edu (A.R.H.); ehelmick@ufl.edu (E.E.H.); bbahder@ufl.edu (B.W.B.); 2 School of Biological Sciences, University of Nebraska-Lincoln, 412 Manter Hall, Lincoln, NE 68588, USA; cclee@huskers.unl.edu; 3 Department of Entomology and Nematology, University of Florida, 1881 Natural Area Dr., Gainesville, FL 32608, USA; hahnp@ufl.edu

**Keywords:** insect vector, phytoplasma, vector preference, interactive effects, Hemiptera, Cixiidae

## Abstract

**Simple Summary:**

Phytopathogen-induced changes often affect insect vector feeding behavior and potentially pathogen transmission. The impacts of pathogen-induced plant traits on vector preference are well studied in pathosystems but not in phytoplasma pathosystems. Therefore, the study of phytoplasma pathosystems may provide important insight into controlling economically important phytoplasma related diseases. In this study, we aimed to understand the impacts of a phytoplasma disease in palms on the feeding preference of its potential vector. We investigated the effects of a palm-infecting phytoplasma, lethal bronzing (LB), on the abundance of herbivorous insects. These results showed that the potential vector, *Haplaxius crudus*, is more abundant on LB-infected than on healthy palms. In contrast, other insects are more abundant on healthy over infected-palms or have no difference between the infection status of palms. Additionally, we further examined the impacts of LB, palm height, temperature, and their interactive effects on *H. crudus* abundance, and the results revealed significant interactive effects of these factors on *H. crudus* abundance. These results suggest the involvement of multiple biotic and abiotic factors influencing vector preference. The results of the interactive impacts of phytoplasma, palm height, and temperature on vector preference in natural settings provide useful information for optimizing vector monitoring and disease management strategies.

**Abstract:**

Insect vector feeding preference and behavior play important roles in pathogen transmission, especially for pathogens that solely rely on insect vector transmission. This study aims to examine the effects of the 16SrIV-D phytoplasma, the causal agent of lethal bronzing (LB) disease of palms, on associated auchenorrhynchan insects. The numbers of auchenorrhynchans collected during weekly surveys during a yearlong study using yellow sticky traps were analyzed. The cumulative number of *H. crudus* was 4.5 times greater on phytoplasma-infected relative to non-infected palms. Other auchenorrhynchans showed no difference between phytoplasma-infected and non-infected palms or were greater on non-infected rather than on infected palms. Furthermore, we examined the effects of LB, palm height, temperature, and the interactive effects of these factors on *H. crudus* abundance. When the palms were infected with LB, at low temperature, *H. crudus* was more abundant on shorter than taller palms; however, *H. crudus* was more abundant on taller than shorter palms at the median and higher temperatures. These results may indicate that *H. crudus* prefers LB-infected palms over non-infected palms. The interactive effects of LB, palm heights, and temperature further suggest that vector monitoring and disease management should be optimized according to seasonal variation in temperature.

## 1. Introduction

Phytopathogens induce morphological and physiological changes in host plants, often with negative fitness consequences. These changes play critical roles in shaping plant-insect vector interactions in the way of favoring pathogen transmission [1,2,3,4,5,6,7]. Plant traits that change due to pathogen infection (e.g., phenotypes and volatiles) also are the cues that are used by insects to orient to their hosts [3,6,8,9,10]. For instance, leaf discoloration is one of the common symptoms displayed on pathogen-infected plants that have influenced the feeding choice of insect vectors [11,12]. Studies showed that discolored leaves of virus-infected plants are preferred by insects when compared to asymptomatic leaves of healthy plants [1,13]. Vector preference for pathogen-infected plants potentially increases the probability of pathogen acquisition by their vectors and may ultimately accelerate disease spread [5,14].

Vector preference of pathogen-infected plants is well studied in plant virus pathosystems, including non-circulatively transmitted viruses (including non-persistent and semi-persistent) [13,15] and circulatively transmitted viruses (including circulative-propagative and circulative-non propagative) [5,8,16,17]. Among cases of persistently transmitted viruses, vectors typically exhibit preference for infected hosts over uninfected hosts, but this is only marginally so among non-persistently transmitted viruses. [4].

Additionally, the preferential response of vectors toward pathogen-infected host plants has also been reported in pathosystems other than plant viruses such as persistently transmitted pathogenic bacteria *Candidatus* Liberibacter asiaticus that causes huanglongbing [18,19] and *Candidatus* Liberibacter solanacearum that causes potato zebra chip disease [20]. Preferential response to pathogen-infected plants is, therefore, considered to be the result of convergent evolution in vectors that share the same transmission mechanisms [5]. The preferential response increases the chance of vector to be in contact with pathogen-infected plants and ultimately favors the transmission of the pathogens by vectors and promotes their spread [4,5].

Phytoplasmas, phytopathogenic bacteria that colonize plant phloem [21], cause diseases that induce changes in plant morphology and physiology that include symptoms of witches’ broom, phyllody, discoloration, and decline [22,23]. Phytoplasma-infection-induced changes of plants presumably affect the cues for insects to orient towards their hosts. Moreover, phytoplasmas are transmitted in a persistent manner [24]. When acquired by its vector, phytoplasmas traverse the midgut, hemocoel and finally invade the salivary glands to be transmitted to the hosts [22]. Based on phenotypic changes of phytoplasma-infected plants and the transmission mechanism of phytoplasmas, we speculate that vectors of phytoplasmas might also prefer phytoplasma-infected plants over non-infected hosts. Additionally, as an increasing number of plant diseases have been discovered to be associated with phytoplasmas, and the economic importance of phytoplasmas is increasing worldwide [25,26], knowledge of phytoplasma transmission by its vector(s) is critical. However, limited research has been conducted regarding vector preference in phytoplasma pathosystems [27,28,29], which will play an essential role in phytoplasma disease epidemiology.

The 16SrIV-D phytoplasma is a palm-infecting phytoplasma that has heavily influenced the natural landscape and palm nursery industry by causing lethal bronzing (LB) disease in various palm species [30]. Reported economic losses due to LB in a local palm nursery were $4.5 million [30]. Symptoms of LB include necrosis of inflorescences, bronze discoloration of leaves, and eventually the death of the entire palm [31,32]. Several insects were proposed as vectors of LB [33,34,35] and, recently, a planthopper, *Haplaxius crudus* (Van Duzee) (Hemiptera: Cixiidae) was identified as a potential vector of 16SrIV-D phytoplasma based on the molecular evidence [36] and transmission assays [37].

In this study, we addressed the impacts of phytoplasma infection on associated auchenorrhynchan insect population abundance using 16SrIV-D phytoplasma-palm pathosystem. All potential vectors of LB phytoplasma are hemipteran insects belonging to the Auchenorrhyncha [33,34,35,36,37]. We hypothesized that the abundance of the potential vector, *H. crudus*, would be affected by LB disease status of palms, and *H. crudus* would be more abundant on LB-infected than on non-infected palms. The non-vector insects would have no such preferential response between LB-infected and non-infected palms. To test the hypothesis, we analyzed the number of auchenorrhynchan insects collected on LB-infected and non-infected palms weekly over a one-year period. Additionally, we further monitored weekly average temperature and palm height to examine possible interactions between these factors and LB on the abundance of *H. crudus*.

## 2. Materials and Methods

### 2.1. Study Site

Surveys were conducted at Fort Lauderdale Research and Education Center (FLREC, Davie, FL, USA; 26°05002.42” N, 80°14014.75” W; approximately 344 km^2^). Lethal bronzing disease has been actively spreading at FLREC since 2014 [38].

### 2.2. Data Collection

**Figure 1:**
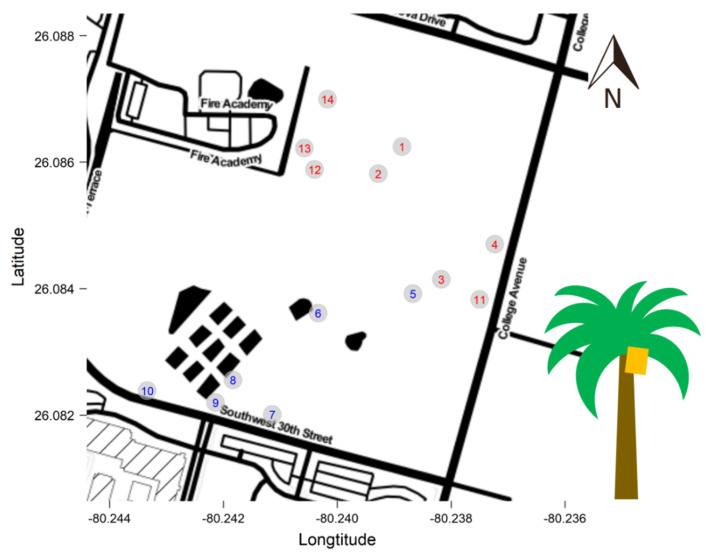
Locations of the palms set up for yellow sticky traps. Lethal bronzing-infected palms (traps 1–4 and traps 11–14) in red and non-infected palms (traps 5–10) in blue.

Eight LB-infected and six non-infected palms were monitored for this study (Figure 1). One yellow sticky trap (Phercon Unbaited AM Yellow Sticky Traps, Gempler’s, Janesville, WI, USA) was placed per palm at canopy level (Figure 1). All traps were collected and replaced weekly. Disease status of the palms used in this study was determined using nested PCR assay with P1m/LY16-23Sr [39] and LY16-Sf2/LY16-23Sr2 [40] primer sets as well as visual inspection of the disease symptoms.

Among the traps, ten traps (trap 1 to trap 10) were collected for a year (October 2017 to October 2018), and four traps (trap 11 to trap 14) were collected for seven months (March 2017 to October 2017). Collected traps were kept in a −20 °C freezer and stored for insect identification. Insects were identified to the species or family level, and the number of each species or family of insects was counted. Four families of auchenorrhynchans (Cixiidae, Cicadellidae, Derbidae, and Membracidae) that were collected consistently by the traps were used in the analysis. Palm heights were measured from the ground to the location of the installed traps (i.e., palm canopies). Weekly average temperatures were calculated according to the trap collecting date using daily average temperature data obtained from the National Oceanic and Atmospheric Administration database (https://www.ncdc.noaa.gov).

### 2.3. Statistical Analysis

Data analyses were carried out in R version 3.6.3. [41]. To assess variations in auchenorrhynchan insect abundance regarding the effects of LB, a generalized linear mixed-effects model (GLMM) was constructed using the glmer function in the lme4 package [42] with a Poisson distribution. In this model, insect abundance was the response variable. Lethal bronzing disease status (two levels: LB-infected or non-infected), insect family (four levels: Cixiidae, Cicadellidae, Derbidae, Membracidae), and the interaction of LB and insect family were predictor variables. Due to the nested nature of our data collection protocol (i.e., multiple palms were resurveyed throughout the study period), insect family was nested within LB nested within trap number as random effects in the model. Overdispersion in the model was corrected by adding an observation-level random effect to the model [43].

The result of the model showed that the abundance of Cixiidae was significantly affected by LB (see Results below). Therefore, another GLMM model was constructed to further assess other variations in the abundance of *H. crudus*, which is the only species belonging to Cixiidae collected in this study. This model also used the glmer function in the lme4 package [42] with a Poisson distribution. In this model, palm heights (continuous variable) and weekly average temperatures (continuous variable) were included in the model in addition to LB status (two levels: LB-infected or non- infected). Additionally, all interactions among these variables were included as fixed effects. Lethal bronzing disease status was nested within trap number as random effects. Overdispersion was also corrected using observation-level random effects [43].

For all models above, analysis of deviance table and *p*-values were calculated using the Anova function in the car package [44]. Means, SE, confidence intervals, and *a priori* linear contrasts were calculated using the emmeans function in the emmeans package [45]. Marginal and conditions *R*^2^ values were calculated using the r.squaredGLMM function in the MuMIn package [46].

## 3. Results

### 3.1. Effects of LB on Insect Abundance

**Figure 2:**
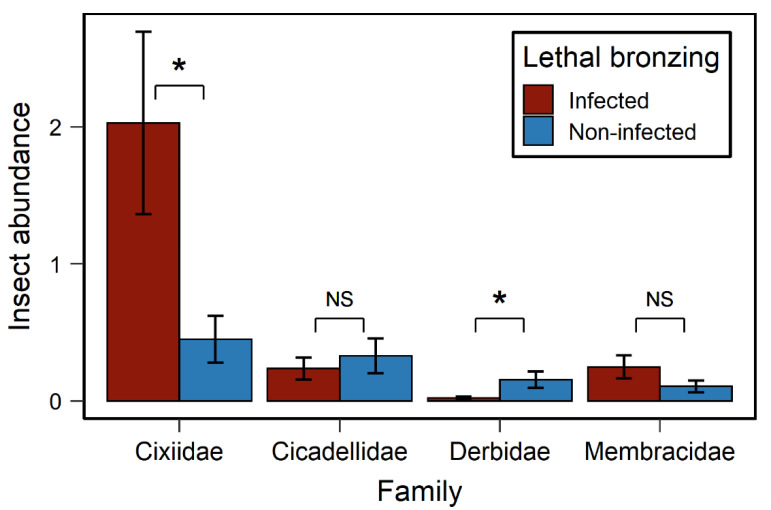
Insect abundance on lethal bronzing-infected and non-infected palms. Values are estimated marginal means ± SE of each family on each trap each week. Asterisks (*) represent significant difference (*p* < 0.05) between LB-infected and non-infected palms under ANOVA of GLMM model.

In total, 3686 individuals of insects belonging to four families in the suborder auchenorrhyncha were collected from the survey and used for the GLMM analyses. Insect family (*χ*^2^ values = 61.2, *p* < 0.0001) and the interaction of family and LB (*χ*^2^ = 24.8, *p* < 0.0001) significantly affected insect abundance, but not LB independently (*χ*^2^ = 0.3, *p* = 0.566). These results indicate that the abundance of auchenorrhynchans were significantly different among families, and that the impact of LB on abundance differed among families. On average, the number of cixiid planthoppers (mean = 0.956 per trap, 95% confidence intervals = 0.585–1.562) was 3.44 to 16.4 times higher than the abundance of other families (*p* < 0.05 for all linear contrasts comparing Cixiidae to the three other families). The abundance of cixiids and derbids was significantly affected by LB (Figure 2). The number of cixiids on LB-infected palms was 4.5 times more than on non-infected palms; the number of derbids was, however, 7.14 times less on LB-infected palms than non-infected palms (Figure 2). In this GLMM, the fixed effects explained 32% of the variation (*R*^2^*_marginal_* = 0.32).

### 3.2. Effects of LB, Palm Height, and Temperature on H. crudus Abundance

The average abundance of *H. crudus* on each trap each week was 2.086 (95% confidence intervals = 0.89–4.8) and 0.519 (95% confidence intervals = 0.18–1.46) on the LB-infected and non-infected palms, respectively (Figure 2). The main effect of LB on the abundance of *H. crudus* was marginally significant, and the average temperature significantly affected the abundance of *H. crudus* (Table 1). Moreover, the interaction of LB × temperature, palm height × temperature, and the three-way interaction of LB × palm height × temperature significantly affected the number of *H. crudus* (Table 1).

**Figure 3:**
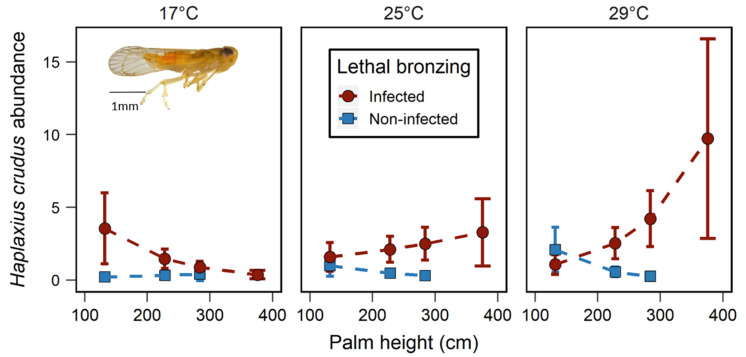
Mean ± SE of *Haplaxius crudus* abundance on lethal bronzing-infected and non-infected palms at different temperatures and palm heights. Dots are estimated marginal means at the lowest (132 cm), the median (228 cm), and the highest (284 cm for non-infected and 376 cm for lethal bronzing-infected palms) tree height.

To visualize the interactive effects of LB × palm height × temperature on *H. crudus* abundance, we calculated estimated marginal means for the two LB treatments at different levels of temperature and palm height (Figure 3). At low temperature (17 °C, the minimum temperature in our dataset), when the trap was installed on short palms (minimum palm height 132 cm), H. crudus abundance was 16.1 times higher on LB-infected than non-infected palms (p = 0.009); at the median palm height (228 cm), H. crudus abundance was 4.6 times higher on LB-infected than non-infected palms (p = 0.057); at high palm height (284 cm, the maximum height of non-infected palms in our dataset), while *H. crudus* abundance was still 2.2 times higher on LB-infected than non-infected palms, the effects of LB status was not significant (*p* = 0.510) (Figure 3). On the other hand, at the median and maximum temperatures (25 and 29 °C), number of H. crudus were significantly greater on LB-infected palms than non-infected ones on the median height palms (228 cm & 25 °C, p = 0.034; 228 cm & 29 °C, p = 0.039) and tall palms (284 cm & 25 °C, p = 0.046; 284 cm & 29 °C, p = 0.011) but not on short palms (132 cm & 25 °C, p = 0.62; 132 cm & 29 °C, p = 0.491) (Figure 3). In summary, during cool temperatures, H. crudus was more abundant on short LB-infected palms, whereas at warmer temperatures, H. crudus was more abundant on tall LB-infected palms. In this GLMM, 28.6% of variation is explained by the fixed effects (*R*^2^marginal = 0.286).

## 4. Discussion

In this study, we monitored 14 palms in an area where LB is actively spreading. We examined the effects of the devastating and economically important palm-infecting phytoplasma disease, LB, on associated auchenorrhynchan insect abundance. Moreover, we further examined the effects of LB, palm height, average temperature, and their interactive effects on *H. crudus* abundance to investigate whether biotic and abiotic factors, other than phytoplasma infection, affect *H. crudus* abundance and possible interactive effects of these factors and LB on *H. crudus* abundance.

Despite the economic importance and widespread distribution of the LB phytoplasma [30], there are a limited number of studies that have been conducted on vector populations over long-term periods [36]. This may be due to the current aggressive tree-removal management practices to control LB in nurseries resulting in inconsistent study conditions. Lethal bronzing infected palms have been removed from the epidemic areas promptly to slow the disease spread. Unlike the situation in nurseries, LB-infected palms at FLREC were preserved for research. Although the number of palms available in FLREC is less than the number of palms in nurseries, FLREC offers a rare opportunity to conduct a long-term survey of insect populations associated with LB without human intervention. This study had to be conducted at FLREC with a relatively small sample size (14 palms) in order to include the seasonal insect population dynamics.

*Haplaxius crudus* was the most abundant auchenorrhynchan collected on the yellow sticky traps in this study (Figure 2). *Haplaxius crudus* is a putative vector of 16SrIV-D phytoplasma that causes LB [36,37]. The abundance of *H. crudus* was, on average, 4.5 times higher on LB-infected palms than non-infected palms. In contrast, other auchenorrhynchan insect abundances showed no difference between LB-infected and non-infected palms or was higher on non-infected than LB-infected palms (Figure 2). The significantly higher number of *H. crudus* on LB-infected palms likely indicates the preferential response of *H. crudus* to LB-infected palms over non-infected palms.

However, due to the nature of the observational experiment design of this study, the actual causation of the greater abundance of *H. crudus* on LB-infected than non-infected palms remains to be determined by experimentation. Potential causes other than LB may also result in the higher *H. crudus* abundance on LB-infected than on non-infected palms. For instance, *H. crudus* might be in higher abundance on LB-infected palms because of other unmeasured variables, such as the difference of surrounding vegetation between the palms, attracting higher numbers of *H. crudus*, resulting in LB infection of the palms, and thus contributed to the pattern observed in our study. Although there are several potential causes that can result in the significantly higher number of *H. crudus* on LB-infected than non-infected palms, based on the knowledge of vector preference for pathogen-infected plants over non-infected plants that were documented in various economically important pathosystems [3,4,27], we suggest that the preferential response of *H. crudus* to LB-infected palms is most likely. Future studies to conduct choice test assays of *H. crudus* between LB-infected and non-infected palms in a controlled setting may provide supplemental information to explain this phenomenon.

On the other hand, the mechanism underlying the potential preference behavior of *H. crudus* towards LB-infected palms is unclear. Pathogen-induced plant volatile changes were suggested as one of the explanations for the vector preferential response to pathogen-infected over non-infected plants. For instance, in *Candidatus* Phytoplasma mali and its psyllid vector (*Cacopsylla picta*) pathosystem, psyllids preferred the phytoplasma-infected more than non-infected apple twig in Y-tube choice test, and the authors further demonstrated that the preferential response is the response to the different volatiles between phytoplasma-infected and non-infected apple trees [27]. Similar results of the preference of insect vectors for volatile of pathogen-infected plants over non-infected plants were also documented in many pathosystems, such as the psyllid vector (*Diaphorina citri*) of *Ca*. L. asiaticus [18,19,47] and aphid vectors of persistently transmitted potato leafroll virus [15,16,48]. These studies suggest that pathogen-induced impacts on volatile cue play an important role for vectors to select their host plants. The preferential response of *H. crudus* to LB-infected palms may also be associated with the change in volatile due to LB phytoplasma infection of palms. Further investigation of the volatile profile of LB-infected and non-infected palms will be beneficial to understanding the interaction of the LB-palm-vector pathosystem and potentially useful for disease management.

The abovementioned studies demonstrate that pathogen-induced plant volatile significantly affects the feeding behavior of vectors; however, this may oversimplify vector feeding preference and behaviors which are part of a complex pathogen-plant-vector interaction. Studies assessing the difference in volatile profiles between infected and non-infected plants are often carried out in laboratory conditions, which exclude many biotic and abiotic factors that may also play essential roles in vector preference and feeding behaviors in nature. Moreover, many biotic and abiotic factors are known to have impacts on insect abundance: biotic factors, such as host plant density and composition, affect insect abundance and dispersal [49,50]; abiotic factors, such as temperature, significantly influence development, phenology, and activity of insects [51,52,53]. Therefore, we incorporated palm height and temperature for GLMM analysis in this study to examine the impacts of these factors on the abundance of *H. crudus*.

The significant effects of temperature on *H. crudus* abundance (Table 1) might be the results of direct effects of temperature on *H. crudus* such as activity [54] and survival or indirect effects via changing palm hosts such as volatile emission [55] or a combination of both. Nymphs of *H. crudus* feed on the roots of grasses and the adults feed on the foliages of various palm species [56,57]. The adult stage is when *H. crudus* can actively transmit the phytoplasma between palms. The developmental time of the nymphal stages may affect adult *H.* crudus abundance and further affect the population size in the area. The developmental time of nymphs of *H. crudus* to adults is faster at 30 °C (52.6 days) than at 24 °C (80.8 days) [58], and the faster developmental time may result in the greater number of adults collected by the traps at a higher temperature than lower temperature.

Moreover, interactive effects of palm height and temperature with LB phytoplasma infection of palms on *H. crudus* abundance were observed (Table 1 and Figure 3). On LB-infected palms, when the temperature was low (17 °C), there were greater numbers of *H. crudus* on shorter palms (132 cm) than the median height palms (228 cm) and taller palms (112 cm). In contrast, at the median and high temperatures (25 and 29 °C), there were greater numbers of *H. crudus* on the median height and taller palms than collected on shorter LB-infected palms (Figure 3). This phenomenon is complex as there are three factors involved in the interaction. As mentioned above, the effects of LB on *H. crucus* abundance could mainly be due to the potential difference of volatile composition between LB-infected and non-infected palms, and how these volatile blends vary with plant height. Temperature also affects the volatile emission of plants with the amount of emitted volatile generally being higher as temperature increases [55]. Insects are strongly affected by ambient temperature, and on a smaller scale, insects often pursue their preferred microclimates by modifying their behaviors [59,60]. Additionally, studies have reported differences in the spatial and temporal microclimate of tree canopies [61,62]. Therefore, it is possible that microclimates may vary between palm canopies of different heights, and the difference of microclimates between palms is critical when considering the feeding behavior and preference of *H. crudus* under different environment temperatures. We speculate that the impacts of LBD × palm height × temperature may have affected *H. crudus* abundance through the changes of volatile compositions, amount of emitted volatile, and microclimates and resulted in the pattern we observed in this study.

Since the transmission of phytoplasma solely relies on the vector, biology and ecology of the insect vector play critical roles in disease spread. A low ratio of *H. crudus* carrying the phytoplasma (0.672%) means that most *H. crudus* that were collected by the traps are non-infected [36]. Therefore, the preferential response of *H.* *crudus* to phytoplasma-infected palms might increase the chance of this potential vector to be in contact with the phytoplasma and be advantageous for the phytoplasma spread. However, as the “vector manipulation hypothesis” proposes, it is often the case that non-infected vectors prefer pathogen-infected plants while pathogen-infected vectors are more likely to be attract to healthy plants [5,29,63,64,65]. A recent study showed that behavioral changes of *Dalbulus maidis*, the leafhopper vector of maize bushy stunt phytoplasma, depended on the phytoplasma infection status of the vector [29]. In that study, phytoplasma-infected *D. maidis* females preferred healthy plants while non-infected females showed no significant preference between phytoplasma-infected and healthy plants [29]. Therefore, the overall preferential behavior of *H. crudus* population to the phytoplasma-infected palms might change toward healthy palms when the number of infected individuals in the population increases. Additionally, as our results suggest that LB, palm height, and temperature affect *H. crudus* abundance interactively, these factors might also affect phytoplasma transmission.

Vector monitoring is essential for disease management by providing insight on the spatial and temporal vector abundance in a given epidemic area. Applying yellow sticky traps is an efficient method often used for monitoring pest populations in the field [52,66]. A comprehensive understanding of factors that influence vector preference behavior, and incorporation of those factors, would be helpful for developing better vector monitoring tactics. Our results suggest that the phytoplasma disease of palms, palm height, and temperature effect the abundance of *H. crudus* interactively. Therefore, establishment of traps for long-term studies to include various temperature conditions and at different heights of palms would provide more information about vector abundance in disease areas. Comprehensive and informative vector monitoring data would efficiently improve disease management program.

## 5. Conclusions

The present study revealed significantly higher *H. crudus* abundance on LB phytoplasma-infected palms than on non-infected palms. In contrast, other auchenorrhynchans were more abundant on non-infected than infected-palms or have no difference between the infection status of palms. These results indirectly support our hypothesis that *H. crudus*, a potential vector of LB phytoplasma, prefers LB-infected palms than non-infected palms but not non-vector insects. Further analysis suggested the interactive effects of LB phytoplasma, palm height, and temperature on *H. crudus* abundance. The impacts of LB were only observed on shorter palms at low temperature, and on the median and taller palms at the median and high temperatures. At the low temperature, *H. crudus* was more abundant on shorter than taller palms; however, *H. crudus* was more abundant on taller than short palms at the median and higher temperatures. The interactive impacts may be related to the volatile difference between palms of different LB infection statuses and palm heights at different temperatures. These results provide preliminary evidence that further study on the volatile of LB-infected and non-infected palms is warranted. Additionally, this study highlights multiple interactive biotic and abiotic factors that affect *H. crudus* abundance. Understanding the factors involved in shaping vector preference and the underlying mechanisms of the interactive effects on vector preference will be essential for future studies of vector biology and for developing disease management strategies.

## Figures and Tables

**Table 1:** Analysis of deviance table of the generalized linear mixed-effects model.

Effects	*χ*^2^ Value	*p*-Value
LBD	2.8	0.097
Palm height	0.5	0.485
Temperature	38.7	<0.0001
LBD × Palm height	1.4	0.242
LBD × Temperature	9.0	0.003
Palm height × Temperature	35.6	<0.0001
LBD × Palm height × Temperature	22.3	<0.0001

*χ*^2^, Type II Wald *χ*^2^ tests.
